# Experience of patients hospitalized with COVID‐19: A qualitative study of a pandemic disease in Iran

**DOI:** 10.1111/hex.13280

**Published:** 2021-07-05

**Authors:** Sara Jamili, Hosein Ebrahimipour, Amin Adel, Shapour Badiee aval, Seyed Javad Hoseini, Marjan Vejdani, Zahra Ebnehoseini

**Affiliations:** ^1^ Student Research Committee Department of health economic and management sciences Mashhad University of Medical Sciences Mashhad Iran; ^2^ Department of Health Economics and Management Social Determinants of Health Research Centre Mashhad University of Medical Sciences Mashhad Iran; ^3^ Health Management and Economics Research Center Iran University of Medical Sciences Tehran Iran; ^4^ Department of Complementary and Chinese Medicine, School of Persian and Complementary Medicine Mashhad University of Medical Sciences Mashhad Iran; ^5^ Department of Medical Technology, School of Medicine Mashhad University of Medical Sciences Mashhad Iran; ^6^ Department of Medical Informatics School of Medicine Mashhad University of Medical Sciences Mashhad Iran

**Keywords:** COVID‐19, Iran, phenomenology, qualitative study

## Abstract

**Background:**

The spread of COVID‐19 as an infectious disease brings about many newly arrived challenges, which call for further research on the scope of its effect on life due to the special conditions of this disease. The present study is, therefore, an attempt to understand the lived experience of inpatients hospitalized with COVID‐19.

**Method:**

In this phenomenological study, among patients with COVID‐19 who were hospitalized in COVID‐19 referral hospitals, 17 people were selected by random sampling method. Data were gathered by interviews and analysed using MAXQDA10 software.

**Findings:**

Analysis revealed 4 main themes and 16 subthemes. Main themes included the (1) denial of the disease, (2) negative emotions upon arrival, (3) perception of social and psychological supports and (4) post‐discharge concerns and problems.

**Conclusion:**

Patients with COVID‐19 experience a different world of stresses, concerns and feelings in the course of their disease. Gaining a deeper insight into patients’ experiences with this disease can help handle this disease more effectively and provide better post‐corona nursing and psychological care and services.

## 1 BACKGROUND

In late December 2019, an unknown pneumonia disease was reported in Wuhan, China, with clinical symptoms highly similar to viral pneumonia, and the World Health Organization (WHO) named the virus COVID‐19.^1^ This is the third kind of coronavirus that has emerged in the past two decades and has caused a multinational outbreak, transmission of complications and considerable mortality.^2^, ^3^

This disease has become widely prevalent in Iran, causing confusion, distress and change in people's living conditions. According to the statistics published by the Ministry of Health and Medical Education of Iran, a total of 361 150 people were infected with this disease by 24 August 2020, and 20 776 of them have passed away.^4^

Despite the specific symptoms of each of these outbreaks (SARS, MERS and COVID‐19), the progressive outbreak of COVID‐19 is causing challenges similar to those of SARS and MERS outbreaks and the lessons learned from those outbreaks could be used.^5^ The emergence of COVID‐19 is currently known as a global health crisis.^6^ A significant deal of anxiety is attached to this disease and leads to changes and a decrease in the life quality of people.^7^ Moreover, due to the high prevalence of this disease, the medical staff is mainly focused on the treatment of this disease and there is less chance to study the psychological conditions of the patients. However, since the course and prognosis of this disease are unknown in different people and there is a lack of adequate information on its nature,^7^ similar to other life‐threatening diseases that reduce life expectancy, it can have important psychological effects such as fear of death, anxiety, depression and stress on the patients and negatively affect the disease course and recovery process.^8^

The importance of conducting psychological and psychiatric interventions for all people affected by large‐scale crises (eg patients and their families, medical workers) has been deemed critical in prior literature.^9^ A review of research in this area showed that the majority of research on COVID‐19 thus far has focused on the epidemiological,^10^, ^11^ pathological and physical aspects of this disease,^12^, ^13^ with relatively little data on direct psychosocial experiences of affected people by the COVID‐19 outbreak. One example of psychological intervention during COVID‐19 is the Chinese Society of Psychiatry (CSP) assigning psychiatrists and psychologists to assist with the integration of psychological interventions with prevention and treatment plans, psychological crisis intervention (PCI) for all affected people, onsite and remote consultation and establishing service prioritization.^14^, ^15^ Based on our literature review and considering the novelty of COVID‐19, no study has been conducted on the experiences of hospitalized patients due to COVID‐19 in Iran and the challenges and problems they are dealing with. Understanding these experiences can help us identify the challenges and problems of patients suffering with COVID‐19 and manage this disease more effectively. Moreover, the results of such research can be helpful for social prevention plans and recovery programmes, which could further contribute to the effective and timely return of affected people to their pre‐pandemic ways of life. The present study is an attempt to explore the challenges and concerns faced by people who have been hospitalized due to COVID‐19.

## 2 METHOD

### 2.1 Participants

This descriptive‐phenomenological study was carried out in 2020. The researchers selected 17 patients from the special ward for patients with COVID‐19 from two COVID‐19 referral hospitals from March to July 2020 by random sampling method based on the following criteria for interview: people who tested positive for PCR and also hospitalized for more than three days in special ward for patients with COVID‐19 (Table 1). The researchers kept samplings until reaching information saturation to eliminate the biased viewpoints and their subjective inferences regarding information saturation and provided the data collected from the participants to two other experts. Sampling continued until the adequacy of information was approved by both of them.

**Table 1:** Demographic data of the 17 patients with COVID‐19

Demographics		Number (%)	Demographics		Mean
Gender	Female	10 (59%)	Age (year)	Total	42.23
	Male	7 (41%)		Female	41.6
Marital Status	Female	Married 8 (80%)	Male	43.14
		Single 2 (20%)	Average length of stay (day)	Total	8.5
	Male	Married 5 (71%)		Female	9.4
		Single 2 (29%)		Male	7.42

### 2.2 Interview Procedure

In the first phase, three in‐depth and unstructured face‐to‐face interviews were conducted to collect data. After data analysis, the interview instructions were developed by the research team and the rest of the interviews were carried out as semi‐structured interviews. The interview guide questions are listed in Box 1. Due to the conditions of the COVID‐19 pandemic, face‐to‐face interviews were not possible, so interviews were conducted by telephone. Before starting the interview, the aims of the present study were clarified to the participant. All interviews were performed by the first author and digitally recorded after obtaining the consent of the subjects. Interviews were conducted from June to September 2020, and the duration of interviews with patients varied from 35 to 70 minutes. The questions were designed to unveil the participants’ experience with COVID‐19. All the patients had mastery of the Persian language and there was no need for translation.

BOX 1 **Interview guide questions**

How did you feel when the first symptoms of the disease started?

How did you react when the doctor said they were suspicious of COVID‐19?

How was your first entry into the COVID‐19 ward?

Given that you could not be accompanied, would not this have been a problem for you?

Did you see the death of the disease up close? How did you feel seeing this scene?

What made you more anxious during your hospital stay?

Who or what helped you calm down more during those difficult days?

What do you think motivated you to be strong and pass this course sooner?

Have you ever wondered if you may have infected someone and blamed yourself for infecting others?

What was the home quarantine era like?

How did people treat you after the quarantine was over and you returned to the community?

### 2.3 Data Analysis

For the data analysis, the recorded data were first transcribed word for word. The data were analysed using the method described by Colaizzi^16^ In the first stage of the analysis, the researcher read the transcribed data several times, focusing on the context of the data and participant responses, and selected significant statements. Then, similar expressions were grouped and organized among the extracted statements, and they were reconstructed in a more abstract fashion. This was followed by the extraction of a theme by grouping similar content in significant statements, and similar themes were grouped and categorized into themes with high abstractness. Data collection and analysis were performed simultaneously. Interviews were conducted till theoretical saturation was achieved such that no new contents appeared in the interviews and the same type of concepts and themes emerged in the data. Data were analysed using MAXQAD10.

## 3 RESULTS

The participants were 17 patients (10 women and 7 men), and their age range varied from 23 to 65 years. A total of 1450 raw codes were obtained. Afterwards, by combining these codes, a total of 16 subthemes or secondary themes were obtained and eventually 4 main themes were obtained (Table 2).

**Table 2:** Main themes and subthemes

Theme	Subthemes
Denial of the disease	Denial of the disease Confusing COVID‐19 with other diseases
Growth of negative emotions upon arrival	Loneliness and lack of a companion along with despair, confusion, and ambiguity
Experience of fear and stress	Fear of corona disease Fear of relapse Fear of the hospital space and environment: Fear of the corona wards Fear of the nurses’ uniforms Fear of experiencing the isolated rooms Fear of experiencing the intensive care units
Death anxiety	Fear of death Fear of seeing other people die Fear of leaving their families alone
Concerns and worries	Concerns about the family members Fear of infecting the family members Fear of the family's status in the absence of the individual Concerns about one's job
Perception of social and psychological supports	Family's emotional support The medical staff's emotional support Religious beliefs Patient's good spirit
Post‐discharge concerns and problems	Escaping and hiding the disease from people Difficulty of meeting the quarantine standards Perception and acceptance of the behaviour and attitude of people

### 3.1 Denial of the disease

The first theme extracted from the participants’ statements was the denial of this disease. This theme consisted of two subthemes, namely the denial and disbelief of the disease and confusion of the disease with other diseases.

One of the participants stated that: ‘*I had a sore throat at the beginning. I thought it was caused by the use of disinfectants and alcohol because the smell of these substances irritates my throat and increases my sore throat frequencies. Therefore, I did not take it seriously at all. I was also very careful and I could not believe I had contracted the corona disease’*. (P8, F, age 36).

### 3.2 Negative emotions upon arrival

#### 3.2.1 Loneliness and lack of a companion

One of the main problems voiced by the participants was the lack of a companion upon arrival at the ward. Many of them stated that they were alone in spite of being in a bad physical condition and had no one to help them take care of themselves.

‘I was very sick and I could barely walk. There was no one to help me and it felt like my world was falling apart. I was very thirsty but there was no one to bring me a glass of water or a cup of tea’. (P16, F, age 61).

#### 3.2.2 Despair

Despite the release of information and news of this disease in the social networks and society, the patients experienced despair upon arrival when they were just trying to accept and believe their sickness.

‘When they were taking me to the ward (Covid‐19 ward (, I thought “this is the end”. I thought I could not be cured and that was the point of no return’. (P7, F, age 43).

#### 3.2.3 Confusion and ambiguity

The complex, ambiguous and unknown nature of this disease affects the patients’ perceptions of the disease, causing anxiety and concern.

‘They would come and give me a shot and a serum injection every day. We felt like laboratory mice. Every doctor would test a drug on us. I did not know what was going to happen. One day a patient would die. The next day another one that was very sick would be discharged fully cured. I did not know the end’. (P15, F, age 58).

#### 3.2.4 Experience of fear and stress

One of the main themes extracted from the interviews was the experience of fear and stress, which is highly common among the patients given the novelty and unknown nature of the disease. This theme included topics such as fear of COVID‐19, fear of relapse and fear of the hospital space and environment.

‘In mid‐February, there was little information on this disease unlike now. People were terrified and I was very terrified too. I was hearing a lot of horrible things about this disease. It was very difficult for me and when I realized I was infected I cried out of terror’. (P5, F, age 37).

‘I was feeling a little better. They brought a very sick patient to our room. We were all terrified of contracting the disease again. We would tell the nurses: “we are almost cured. Why did you bring them here to infect us again?” My whole body would shake every time the patient coughed because I was terrified of being infected again and going through all that pain and difficulty once more. I am still the same and if someone coughs near me, I am terrified of experiencing those difficult times again’. (P2, F, age 43).

‘When I was in the ICU, I looked at the patients on the other beds and I was even more terrified. The sound of their devices was very scary. I thought someone was about to die. I had heartache because of the fear, I swear. I thought it was all because I was in that ICU ward. I constantly thought “God am I going to be like them? Will I be so sick that they have to put these tubes in my throat?” I thought they took me there because I was so sick’. (P3, M, age 32).

#### 3.2.5 Death anxiety

One of the primary themes extracted from the interviews was the anxiety and fear of death theme, which consisted of three subthemes namely the fear of death, fear of leaving one's family alone and fear of seeing the death of another person.

Death anxiety is a complicated theme that consists of the fear of one's death and others’ death.^17^

‘It was very difficult. The eyes of the patient next to me were open early in the night and he was awake. A few hours later, he died suddenly. He got short of breath and died before my eyes. I was terrified seeing him die and I thought “God who knows what will happen to us. I do not know if I will be alive a few hours later or not. Perhaps I will not wake up in a few hours like this man.” I had never felt death was so close’. (P11, F, age 43).

#### 3.2.6 Concerns about the family members

Another concern of these patients was that they were unsure whether they had infected their family members because they had contact with their family members prior to hospitalization and confirmation of their sickness. They also felt guilty for transmitting this infection to others. For instance, (P8, F, age 36) said: ‘*I was mostly worried about my family because it was during* *Nowrooz days) is the Iranian New Year (and we had gone to my parents’ and my father‐in‐law's* *houses. I had also visited some other people. I thought since I had COVID‐19 and I visited all those people; I might have infected them too. My parents are very old and suffer from diabetes and high blood pressure. What if I infected them too?’* (P11, F, age 43).

#### 3.2.7 Concerns about one's job

Since most of the patients had to leave their workplace for more than two weeks due to the long‐term hospitalization that usually lasted for more than 5 days and since they had to be in quarantine afterwards, they were worried about their jobs.

‘It was the end of the year and I had many checks and installments. I was worried about paying all of them. I had asked four young workers to run my workshop and I was worried about them making a mistake and creating a huge problem. I thought how I could satisfy my clients’. (P14, M, age 39).

### 3.3 Perception of social and psychological support

#### 3.3.1 Perception of the family's emotional support

Perceived support refers to the subjective perception of the availability of support, adequacy of support and the quality of support in states of emergency.^18^, ^19^ The family was the first and the most important source of social support for the patients, which motivated them to fight the disease and the resulting stresses.

‘I do not know if I could have survived if it were not for the phone calls made by my family members. I would feel less stressed every time I talked to them and heard my children's voices’. (P1, F, age 35).

#### 3.3.2 Social support of the medical staff

The kind and empathetic attitude of the nurses and the other medical staff members improved the spirit of the patients fighting this disease.

‘The nurses would talk to me a lot because I stayed in the hospital for a long time. I was mentally weak and one day I cried and asked why I was not getting better. One of them came to me and talked to me a lot. She said: “You should thank God that you are young and you will recover. You were very sick but your condition is improving every day. You will be discharged soon.” Her words really made me feel better’. (P6, M, age 28).

#### 3.3.3 Religious beliefs

The belief in God empowers the patients, and people believe that God is their protector and guide. They believe God's support enables them to defeat their disease.

‘I only resorted to God. I knew if he wanted me to live, diseases worse than corona could not defeat me’. (P16, F, age 61).

### 3.4 Post‐discharge concerns and problems

#### 3.4.1 Avoiding people and hiding the disease from people

The last theme was the post‐discharge concern of the patients after their return to society. Fear of stigma had made them hide their disease from others. People's avoidance of the patients, rejection from society and people's attitude to the patients were among the behaviours that were more difficult to handle than the physical pain.

‘I did not want anyone to know because I was worried about my children. I understand if some people avoid me but the children are little and may not be able to understand it. I mean I was scared of people treating my children differently when it was revealed to them. For example, I was worried the neighbors would prevent their children from playing with my kids if they knew’. (P9, F age 44).

#### 3.4.2 Perception and acceptance of the behaviour and attitude of people

Some of the interviewees stated that they had accepted the conditions of their disease and understood people's attitude.

‘I was not hurt by their behavior and perhaps I would also avoid someone with COVID‐19 if I were them. Everyone is worried about their health. I completely got it’. (P15, F, age 58).

#### 3.4.3 Difficulty of meeting the quarantine condition

One of the problems of these individuals was meeting the quarantine conditions after discharge, which had resulted in stress for these patients due to the presence of their families and their fear of infecting their other family members.

‘*It was more difficult for my children. I was hospitalized for 10* *days* *and I was not with them. Afterward, I went home and stayed in my room. My little son was complaining a lot because he is very attached to me. He said: “Mom will return and hug me*”’ (P1, F, age 35).

## 4 DISCUSSION

The themes were extracted from the interviews in an attempt to meet the primary goal of this study, that is unveiling and interpreting the lived experience of patients hospitalized with COVID. This study was carried out to gain a deeper insight into this phenomenon. Our findings are summarized into four themes: (1) denial of the disease, (2) negative emotions upon arrival, (3) perception of social and psychological supports and (4) post‐discharge concerns and problems.

The first experience of people with this disease is related to the onset of the first symptoms of the disease. Due to this pandemic, there are many news and stories as well as considerable fear and anxiety in society in connection with this disease. As a result of this fear, people use mental defence mechanisms such as denial in their first encounter with the symptoms of this disease. Freud defines these defence mechanisms as mental functions that protect people from excessive anxiety caused by external stressful events and internal destructive states.^20^ Denial has an inverse relationship with acceptance and prevents the individual from making the minimum possible effort to solve the problem. This mechanism is used for many different medical diseases and is linked to delays in taking measures to receive treatment and meet a doctor.^21^ As for this disease, most participants stated that they tried to deny and ignore the disease due to fear of this disease and the fear of accepting and believing in this disease. They introduced this factor as the reason for trying self‐medication and visiting medical centres with delay, which exacerbated the condition and led to their hospitalization.

The second category of experiences included the experiences at the time of arrival at the hospital as a patient with COVID and during hospitalization. This category included negative emotions such as loneliness, despair, confusion, ambiguity, fear, anxiety and different concerns of the patients. These results are in line with the study of Sun et al, which states that fear, denial and stigma during the early stages of disease.^22^

The experience of fear and stress are among the main themes reported by the patients. The other themes were either derivatives of fear or causes of fear. Some of the different symptoms of the disease include anxiety and fear, which are common among patients with chronic respiratory disorders such as COVID‐19.^23^ These results are consistent with the study by Alipour et al, who identified psychological distress as one of the main issues in COVID's psychosocial challenges and concerns.^24^

The results of numerous studies of patients with COVID in China during the spread of this disease revealed that some of these psychological disorders such as anxiety, fear, depression, mood changes, insomnia and post‐traumatic stress disorders have been reported with a high prevalence rate among these patients.^25^, ^26^ The uncontrolled spread of COVID‐19, the unsatisfactory condition of patients isolated in the intensive care units with acute respiratory diseases, the lack of an effective drug and the mortality caused by this disease are among the most important factors that can affect the mental health of patients infected with this disease.^27^, ^28^, ^29^ According to the research results,^30^ these patients have low psychological tolerance, and considering the current status of the disease in the world, these individuals are highly prone to mental disorders such as anxiety, fear, depression and negative emotions as confirmed by the results of the present study.

Death anxiety is also a complicated concept that cannot be explained easily, and it generally consists of themes such as the fear of one's death and others’ death. A study carried out on the experience of death anxiety in COVID patients reported the following themes as the subthemes of the experience of death anxiety^8^ : a mental obsession with death, the fear of the funeral quality, the inability to see one's loved ones before death, watching one's loved ones die, having dreams about death, ambiguities about COVID deaths and death anxiety with exacerbated physical symptoms, especially dyspnoea.

As patients with a life‐threatening disease grow closer to death, the role of social support, including the family members, increases while the presence of the family members reduces death anxiety in the patients.^36^ However, these patients were not allowed to see their families, friends and relatives during hospitalization and this factor increased death anxiety in them.^31^

In the present study, patients of different age and gender groups had different feelings about death anxiety. Older patients had less anxiety about death, but younger people had higher anxiety, and seeing the death of other patients had a profound effect on them and made them see death very close to them. However, in response to the question about how they overcame their stress and anxiety, most of them stated that the anxiety could be overcome by dint of the mental and spiritual support of the families and the medical staff along with one's religious beliefs and trust in God. (Iran is a Muslim country and its people strongly believe in the existence of God.)

Another motivating force that enabled people to overcome these negative emotions was perceived social support from family and medical staff. Family is an important and fundamental part of an individual's health and can play a significant role in the patient's health.^32^ Research results suggest that the presence of family members significantly mitigated the patients’ anxiety.^33^ However, in the COVID pandemic, the presence of the family by the patient's side is not possible due to the high rate of the outbreak and the risk of transmission of the disease, and thus, the family is kept away from the patient. A COVID patient enters a hospital when it is not known whether they will die or live. The patient cannot see their family throughout the entire hospitalization period. Various studies on other diseases show that when the patient is hospitalized and experiences an unknown environment, being away from one's family, loss of one's professional function and exposure to the unknown processes and tools can lead to the emergence of negative emotions such as stress and anxiety in the patient. Due to the physical absence of the patient's family members and their lack of presence in the hospital, which can hinder the satisfaction of the patient's fundamental need for communication in the stressful condition of the disease, the social support of the hospital personnel sets the scene for the satisfaction of this fundamental need.^8^

No study has been conducted so far on the effect of the absence of the family from the patient's side but studies of patients in the intensive care units revealed that the presence of the family members is forbidden in these wards, especially due to its structure and philosophy, while visiting the patient is highly restricted. This is because nurses believe that the presence of the family increases infection. However, a quasi‐experimental study of the control group showed that the presence of the family members through scheduled meetings significantly reduced the patient's stress as compared to the no meeting group.^33^ Perceived social support was more important among these patients as they were not allowed to meet their family members as long as they were hospitalized in the wards. Social support is a major facilitator of psychological well‐being in stressful conditions and the important evolutions and transitional stages of life. Although stressful events such as contracting a dangerous disease can have destructive effects on the psychological health of individuals, social support serves as a shield protecting the patient from the stress caused by the disease and mitigating its negative effects.^34^

Studies have even indicated that the patients’ mortality rate decreased along with the onset of the physical and psychological conditions with an increase in the perceived social support.^35^, ^36^ Religious beliefs also lead to the emergence of beliefs in peace and God's rewards after death due to the pain of the disease. Spiritual and religious attitudes, which have effective influences on the individuals’ approach to the inevitable truth of death, mitigate the death anxiety by redefining death and enabling the patient to successfully cope with the disease‐related experiences.^37^

Research results revealed that several factors, especially spiritual experiences, positive thinking and perception of social support, influence the patients’ viewpoint on this disease and their attitude to this disease in the diagnosis, treatment and hospitalization stages and increase the level of adjustment to the disease conditions. Spiritual and religious experiences, as a powerful source of adjustment, optimism, hopefulness and meaningfulness, enable the individual to reduce the patients’ bitter experiences with the disease. Resorting to religious and spiritual beliefs, surrendering to God's divine will and trusting in God's grace are considered important and promising sources of overcoming the disease and the uncontrollable conditions as well as means of confronting and accepting the concept of death.^8^

The next sub‐theme extracted from the participants’ experiences included the concerns that occupied the inpatients’ minds during hospitalization and evoked stress and anxiety in them. Most of these patients were worried about their children and old parents and blamed themselves for their failure to meet hygiene standards. They stated that they were only worried about their families' health. In line with this finding, Rahmatinezhad's research showed that the patients found themselves in an uncontrollable and difficult condition with no control over it. Since the patients felt they had no control over their life events and the future of their families sounded vague and they were worried about the transmission of the disease to their family members, including their children they were mainly prone to the feeling of helplessness, despair, distress and anxiety.^8^ Another concern of these patients was economic problems and concerns about their jobs as they were worried about losing their job due to their long‐term hospitalization and quarantine. In this regard, the findings from the study by Mihashi showed that the most common mental problem in China following the recovery from SARS was the concern about the decrease in financial resources and income of households. As a result, this factor was introduced as the most important predictor of reduced mental health.^38^ Moreover, in his study, Khodabakhshi explored the economic concerns regarding COVID‐19 and stated that one of the major concerns of freelancers who stay home was the subsequent financial consequences and problems.^39^

The last group of experiences of COVID‐19 patients includes their post‐discharge experiences when they return to society. Many patients stated that people's treatment of them including avoidance, bad reputation and lack of social interactions even after complete recovery leads to an experience that is more difficult than the physical pain of the disease. This is a phenomenon that can be called the stigma of COVID‐19.

Stigma has always been one of the social challenges in social studies of health, disabilities and mental diseases.^40^ Generally, the social complications of stigma have a devastating effect on health, though the process differs from disease to disease. ^41^ The results of this study suggested that the social stigma related to COVID‐19 caused additional psychosocial tensions and social barriers as well. Stigma can be an obstacle to behaviours of health care seeking and often delays the diagnosis and treatment of disease. Stigma can lead to poverty and ignorance through social marginalization, resistance against health institutions and pervasive distrust.^42^ The results of this study are in line with the results of Alipour et al as well as with results of Rahmatinejad et al^8^, ^24^

To the best of our knowledge, the present study is one of the first qualitative studies on patients' lived experiences with COVID‐19. Despite the novelty of the study and the richness of the data extracted from it, many limitations must be considered. There have been few studies on psychosocial concerns of COVID‐19 and there is not much literature that can be trusted. Researchers have tried to fill this gap to some extent by using the literature of other epidemics and other countries. The present study discusses people's experiences with COVID‐19 but it is limited to independent adults, and the findings may not be generalized to other subsets, such as the elderly, children, and people with disabilities. To better understand the psychosocial challenges, it is suggested that you do more studies for these groups. To better understand psychosocial challenges, it is recommended to perform further quantitative studies that compare the different responses of these groups. Samples were also collected from two hospitals referring to COVID‐19. Because some patients' experiences are due to the hospital environment, the results may vary from hospital to hospital. In this regard, it is suggested that comparative studies be conducted on the impact of the hospital environment on the experiences of such patients.

## 5 CONCLUSION

Patients with COVID‐19 experience a different world of stresses, concerns and feelings in the course of their disease. Most of them had a lot to say and an analysis of their statements can perhaps be effective in providing better services as well as better nursing and psychological care after COVID‐19. Therefore, these people need to receive medical services to cure their physical disease, while they also need psychological interventions because some of the painful mental and psychological experiences of these patients can delay and hinder their recovery. Furthermore, the use of psychological interventions can reduce anxiety and depression in COVID‐19 patients based on the results of this study. Moreover, the findings from this study can be used to introduce the patients’ experienced feelings to the nurses and the medical staff and inform them of the subtle considerations about the patients, thereby paving the way for the fight against this disease.

## DATA AVAILABLY STATEMENT

The data that support the findings of this study are available on request from the corresponding author. The data are not publicly available due to privacy or ethical restrictions.

## CONFLICT OF INTEREST

All authors declare no conflict of interest.

## AUTHORS’ CONTRIBUTIONS

Dr Ebrahimipour designed this study. Jamili, Adel and Vejdani collected the information. Jamili and Ibn Hosseini have been in charge of statistical analysis. Funding, support and supervision were done by Dr Badiei and Dr Ebrahimipour. The original version was also written by Jamili.

## ETHICAL APPROVAL

The procedures followed in the present study caused no physical or psychological harm to the subjects. At the beginning of the interview, the purpose of the study and data confidentiality were explained to the participants and verbal consent was obtained for face‐to‐face and telephone interviews, respectively. The subjects had the right to withdraw from the study at any time. The study has been approved by research committee of Mashhad University of Medical Sciences (code: IR.MUMS.REC.1399.254).
